# Bio-Guided Optimization of *Cystoseira abies-marina* Cosmeceuticals Extraction by Advanced Technologies

**DOI:** 10.3390/md21010035

**Published:** 2022-12-31

**Authors:** Gonçalo P. Rosa, Andreia F. Peixoto, Maria Carmo Barreto, Ana M. L. Seca, Diana C. G. A. Pinto

**Affiliations:** 1 LAQV-REQUIMTE, Department of Chemistry, University of Aveiro, Campus Universitário de Santiago, 3810-193 Aveiro, Portugal; 2 cE3c—Centre for Ecology, Evolution and Environmental Changes, Azorean Biodiversity Group, CHANGE—Global Change and Sustainability Institute, Faculty of Sciences and Technology, University of the Azores, 9500-321 Ponta Delgada, Portugal; 3 LAQV-REQUIMTE, Department of Chemistry and Biochemistry, Faculty of Sciences, University of Porto, 4169-007 Porto, Portugal

**Keywords:** *Cystoseira abies-marina*, *Gongolaria abies-marina*, ultrasound, microwave, cosmeceutical potential

## Abstract

*Cystoseira abies-marina* (reclassified as *Gongolaria abies-marina*) is a brown seaweed species rich in meroterpenoids, presenting interesting antioxidant, antitumor, and anti-inflammatory activities. However, there is still a lot to uncover regarding the bioactive potential of this species, as evidenced by the lack of records of antiaging activities from *Cystoseira abies-marina*, making this macroalga an excellent candidate for studies of its cosmeceutical potential. Ultrasound-(UAE) and microwave-assisted extraction (MAE) are advanced sustainable technologies that are very efficient in enhancing bioactive compound extraction. Applying these extraction techniques to a new biological matrix often calls for optimizing the parameters toward the best extraction yield. Since *Cystoseira abies-marina* is a new matrix for both UAE and MAE techniques, the present work proposes the optimization of the extraction process, using a novel approach: instead of only focusing on increasing the yield, the goal of this work is to determine the parameters for UAE and MAE that lead to extracts with better antiaging activities. For this bio-guided approach, several *Cystoseira abies-marina* extracts were prepared by UAE and MAE under varying conditions of solvent, time, and algae/solvent ratios. Their antiaging activities were then determined, and all the results combined to unveil the conditions yielding extracts with higher cosmeceutical potential. Using statistical tools, it was found that, for UAE, the best conditions were ethyl acetate, 15 min, and a ratio of 1:4, which led to an extract with high yield, and causing the strong inhibition of tyrosinase and elastase. In turn, ethanol, 10 min, and a ratio of 1:4 were the best conditions for MAE, leading to the extract with the best antioxidant activity. The results show that the proposed bio-guided approach was effective in obtaining extracts with high cosmeceutical potential, unveiling the possibility of modulating an extract’s activity by changing the extraction method.

## 1. Introduction

The oceans contain a vast array of organisms that are known to produce a rich collection of distinct metabolites, many of them with novel chemical structures and interesting biological properties. In fact, the exploration of aquatic regions has led to the discovery of more than 10,000 metabolites, many of which have pharmacological potential [1]. 

Among marine organisms, macroalgae are great contributors to the variety of novel compounds of biological interest, mainly promoted by the conditions they endure in their habitats. In fact, because of their sessile nature, these organisms are highly exposed to abiotic (high variations in salinity, temperature, and UV radiation) and biotic (herbivory, bacteria, fungi) stress, which leads to the production of secondary metabolites with a wide range of possible applications [2,3]. The metabolites isolated from macroalgae are documented to possess a variety of biological activities, such as antioxidant, antimicrobial, anticancer, anti-inflammatory, antidiabetic, and antiobesity, with applications as pharmaceuticals, nutraceuticals, cosmeceuticals, and in agriculture [4,5,6,7,8].

Most cosmetic products currently available on the market are still composed of synthetic chemicals, which usually results in a more expensive product that can have nefarious side effects for the consumer. Increased consumer awareness of these disadvantages has led to an increasing preference for products of natural origin. Thus, macroalgae, as natural sources of such a wide variety of bioactive compounds, are a significant target of studies regarding their potential cosmeceutical applications [9].

*Cystoseira abies-marina* (S.G.Gmelin) C.Agardh is a brown macroalga found in the Mediterranean, the Macaronesian Region, and the coast of Africa, which was reclassified in 2020 as *Gongolaria abies-marina* (S.G.Gmelin) Kuntze [10]. However, since this change is very recent and the main studies for this species have been published under the name *Cystoseira abies-marina*, in the present work, we maintain this designation. The meroterpenoids isolated from these macroalgae showed antioxidant activity and high cytotoxic potential against Hela cells [11], suggesting an interesting pharmacological potential. Furthermore, other compounds have been isolated from this species, such as cystomexicones A and B [12] or 1′-methoxyamentadione [13]. With the exception of Sánchez-Camargo et al. [14], all studies with this macroalgae were carried out using classical extraction methods. Additionally, the antiaging properties of this macroalgae are highly underexplored, as highlighted by Rosa et al. [15], making it an ideal target for assessing cosmeceutical potential.

As referred above, the extraction of *Cystoseira abies-marina* mainly used classical methods. However, due to the increasing importance of sustainability in every aspect of daily life, methodologies capable of decreasing the impacts of the extraction process should be emphasized. In recent years, several advanced techniques have been employed to extract biological matrixes, with proven efficacy and reduced environmental impact compared with conventional methods [16]. These could be useful tools for exploring the cosmeceutical potential of *Cystoseira abies-marina*

Ultrasound-assisted extraction (UAE) is one of these examples, which operates based on the phenomena of cavitation and oscillation [17]. When ultrasonic waves are formed within the extraction solvent, their energy is transferred to the solid particles immersed in the fluid. Furthermore, cavitation bubbles are formed closer to the solid surface and then collapse at higher amplitude, resulting in the rupture of cell walls, and thus accelerating the transfer of compounds from inside the cell to the solvent [18,19]. Compared to conventional methods, UAE presents several benefits, such as a higher extraction efficiency, the requirement of a simple instrument setup, less solvent, low energy throughput, and lower extraction times. Furthermore, milder operating conditions prevent the deterioration of less stable compounds [20].

Another advanced technology capable of increasing the efficacy and sustainability of the extraction process is microwave-assisted extraction (MAE). In this method, samples are irradiated with microwave radiation, which causes them to heat by two phenomena that contribute to the extraction process. The first one is dipole rotation, which consists of the alignment of solvent molecules in the direction of the electric field, leading the molecules to rotate at high speed, which creates heat that disrupts cell walls. The second phenomenon is ion conduction, in which the dissolved ions migrate, increasing the penetration of the solvent into the matrix and thus facilitating the collection of the target compounds [21]. Furthermore, the generated heat leads to a buildup of pressure in the system, which modifies the physical properties of the matrix, resulting in higher porosity and swelling of the cell, and allowing for better penetration of the extracting solvent [22]. The main advantage of this technique comes from the possibility of rapidly heating the sample, which allows for a significant reduction in the extraction time and amount of solvent used, leads to improved yields, and is suitable for thermolabile substances [23].

Besides the advantages of using UAE and MAE in recovering bioactive compounds from biological matrixes [24], there are, to the best of our knowledge, no reports of their application to the extraction of *Cystoseira abies-marina*. That knowledge gap, allied with the inexistence of studies about the cosmeceutical potential of *Cystoseira abies-marina,* provides an excellent opportunity to explore both these aspects at the same time. To achieve this, the present work proposes a bio-guided approach to optimization of the extraction parameters for UAE and MAE of *Cystoseira abies-marina*, focusing on the generation of extracts with improved antiaging activities.

## 2. Results and Discussion

### 2.1. Ultrasound- and Microwave-Assisted Extraction

To achieve the optimal extraction conditions to generate extracts with the best antiaging potential, several *Cystoseira abies-marina* extracts were obtained by UAE and MAE, varying the experimental conditions. The extracts were obtained from three solvents with different polarities: hexane, ethyl acetate, and ethanol. This range of polarities was selected to widen the range of extracted compounds and to understand whether the cosmeceutical potential is more significant in polar or non-polar extracts. Two different extraction time conditions and solid/liquid ratios were also evaluated to assess the effect of these operational conditions in an extraction scaling-up without affecting antiaging activity potential. The yields obtained by UAE and MAE extraction processes are shown in Table 1 and Table 2, respectively.

From the results presented in Table 1, it is possible to observe that, in general, extraction yields are higher with polar solvents (ethyl acetate and ethanol). In fact, the yields obtained with hexane using a solid/liquid ratio of 1/4 are lower (<1 mg extract/g d.w.), independently of reaction time (15 or 45 min). However, an exception was observed, improving the liquid fraction (solid/liquid ratio of 1:10) under the longer extraction time of 45 min, leading to Y_ext_ = 6.9 mg/g d.w. (UAE-8). When using ethyl acetate as solvent, the yield of extraction was higher at a lower extraction time (UAE-1, Y_ext_ = 10.0 mg/g d.w.) than for a longer extraction of 45 min (UAE-2, Y_ext_ = 6.5 mg/g d.w.) using the same s/l ratio of 1/4. This shows that, with this solvent, it is possible to achieve a compromise in terms of sustainability, since a shorter time represents less energy consumption being wasted in the process. 

The results presented in Table 2 show that, similarly to the observed for UAE, higher yields were also obtained with polar solvents in MAE. 

In fact, the yields obtained with hexane were low when using microwave-assisted radiation, probably due to the low dielectric constant of this solvent, in accordance with other results reported in the literature [25]. Extract yields were generally higher with 20 min of extraction, with a few exceptions, such as MAE-1, MAE-3, or MAE-9. The highest yield was obtained with ethanol when the solid/liquid ratio was 1:10 and, after 20 min, the Y_ext_ = 7.80 mg extract/g d.w. (MAE-12) vs the Y_ext_ = 2.76 mg extract/g d.w. (MAE-10). 

These results provide an interesting overlook about the effect of operational conditions variations to achieve higher extraction yields. However, since the main goal is to optimize extraction yield and the conditions that lead to extracts with the best anti-aging potential, these data must be crossed with the biological activity determinations.

### 2.2. Antiaging Activities

The aging process can be characterized by intrinsic and extrinsic aging. Intrinsic aging is generally regarded as the average decline associated with the natural process of aging over time. It is affected by the natural deterioration of physiological functions determined by genetic and metabolic factors, without the influence of the environment [26,27]. Conversely, extrinsic aging refers to all aging caused by environmental exposure, lifestyle, and habits. Exposure to sunlight and pollution, tobacco consumption, diet, excessive stress, lack of sleep and exercise are considered aggravating factors for aging [28]. Among these factors, exposure to UV radiation is one of the main contributors to the aging process. It causes acute stress responses, mainly by ROS production, which then leads to the upregulation of extracellular matrix-degrading enzymes and pro-inflammatory mediators, and chronic damage responses, caused by the accumulation of damage in non-proliferating skin cells [29]. Strategies to counteract extrinsic aging, specifically its more visible effects on the skin, often resort to compounds with antioxidant activity due to their capacity to reduce ROS, thus limiting their nefarious effects.

In this regard, antioxidant activity was determined to assess the cosmeceutical potential of the obtained extract. The extracts’ total phenolic content (TPC) was also determined since this family of compounds is known for their high antioxidant activities. The results for antioxidant activity and TPC are detailed in Table 3.

Overall, the antioxidant potential of samples was low, particularly on the DPPH assay, with all the extracts presenting a higher EC_50_ than the maximum tested concentration. Better results were obtained in the ABTS assay, with MAE-2 being the most active extract. However, this activity is 33.3 times higher when compared to Trolox, the positive control, which is not a very promising result. Other samples with better activity correspond to ethanol extracts, which is in line with the results obtained for other brown macroalgae, where the authors found that ethanolic extracts present significantly high antioxidant activity [30,31,32]. Ethanol extracts of other brown algae have also been reported to possess high antioxidant activities [24,33]. 

Comparing UAE with MAE, it is possible to observe that the latter leads to extracts with better antioxidant potential, whereas extracts obtained with UAE have a lower antioxidant activity and higher TPC. Other works have reported on the better antioxidant activities obtained by MAE of brown macroalgae [34,35] when compared to UAE; however, on those cases, the TPC was also higher, contrary to that observed in this study. This means that, for *Cystoseira abies-marina*, MAE favors the extraction of compounds other than polyphenols, which are stronger antioxidants than the phenolics extracted with UAE. This observation is not new in this species. In a previous work, where *Cystoseira abies-marina* was extracted by conventional methods, the extract with a lower TPC was also the one with a better activity on the DPPH assay [36].

Besides directly tackling ROS, when fighting extrinsic skin-aging, it is important to also tackle the increase in the activity of extracellular matrix-degrading enzymes caused by these pro-oxidant molecules. When over-activated, these enzymes excessively break down important structural molecules, such as elastin, collagen, or hyaluronic acid, leading to the formation of wrinkles, loss of elasticity and dryness of the skin, among other effects [37]. A product capable of inhibiting the activity of enzymes such as elastase, collagenase, tyrosinase and hyaluronidase has high cosmeceutical value. 

To assess the antiaging potential of the extracts prepared by UAE and MAE, their capacity to inhibit the enzymes tyrosinase, elastase, collagenase, and hyaluronidase was measured. The results for enzyme inhibition are detailed in Table 4.

The anti-hyaluronidase activity of all 24 tested extracts was 0%, so the results are not included in Table 4. The extracts showed low collagenase inhibitory activity, with none of the extracts reaching 50% inhibition at the maximum tested concentration, therefore preventing the determination of their IC_50_. UAE-1 and UAE-6 extracts were the most active collagenase inhibitors, with all inhibiting more than 35% of activity. Higher inhibitions were obtained with UAE extracts than with MAE extracts.

Regarding elastase inhibition, the results are more positive, with UAE-1 and UAE-3 being the most active extracts (Table 4). The IC_50_ obtained for these two extracts, namely, UAE-1 with 45.4 µg/mL, is a very good result when compared with the literature, since the most recent data on elastase inhibition for macroalgae extracts reported that the best extracts presented an IC_50_ of 209.56 µg/mL [33]. Contrary to that observed for antioxidant activities, where the ethanolic extracts had better activity, ethyl acetate extracts were more active for elastase inhibition, which shows that the extraction conditions can be modulated according to the target activity.

Tyrosinase inhibition was the assay for which the best overall results were obtained. In fact, within the 24 tested samples, more than half inhibited 60% or more of the tyrosinase activity at the maximum tested concentration (Table 4), which shows the potential of *Cystoseira abies-marina* as a source of antiaging compounds.

The most active extract was UAE-1, with an IC_50_ of 7.5 µg/mL, a value that is only 4-fold higher than the obtained for kojic acid, a commercially used tyrosinase inhibitor. This is a very interesting value, as UAE-1 is still a crude extract. Compared with values for tyrosinase inhibition reported for crude extracts of other brown macroalgae in the literature [33,38,39], UAE-1 is the extract with best inhibition reported to date. Some examples are the methanolic extract of *Ecklonia stolonifera*, which presented an IC_50_ of 345 µg/mL [38], a value that is 46 times higher than the obtained for UAE-1. An ethanolic extract from *Turbinaria conoides* showed an IC_50_ of 188.85 µg/mL [39], while an extract of mixed composition obtained from a beach cast had 15.18 µg/mL [33]. Other extracts must be highlighted, namely, UAE-8, UAE-11, and MAE-9, since their IC_50_ values are lower than 50 µg/mL.

Contrary to the observed antioxidant activity, these results show that better enzyme-inhibitory activities are obtained with UAE, in addition to the activities presented by MAE extracts. This is a very interesting observation, since it allows for modulation of an extract’s activity by changing the extraction conditions according to the intended target. 

**Figure 1:**
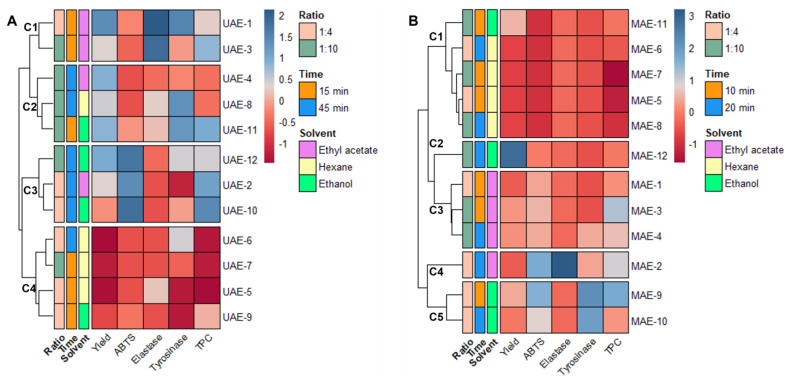
Data matrix obtained by combining the results of extraction and antiaging assays. The heatmap is organized by rows according to the Ward clustering algorithm (Euclidean Distance): (**A**) heatmap for ultrasound-assisted extraction; (**B**) heatmap for microwave-assisted extraction.

As stated at the end of Section 2.1, the data obtained from the extraction must be combined with the results from the antiaging activity to achieve the optimal conditions to obtain *Cystoseira abies-marina* extracts with high cosmeceutical potential for each of the extraction methods. To better understand the effects of each variable in analysis, all variables were autoscaled; then, the relationships between them were visualized through a heatmap, combined with hierarchical clustering (Figure 1).

The DPPH and collagenase variables could not be included in this analysis because their IC_50_ were higher than the maximum concentration tested for all the samples.

Observing the heatmap obtained from the UAE data (Figure 1A), four clusters are evidenced, with samples grouped according to their score for the different variables. One of these clusters (C4) comprises the samples with low yields and poor antiaging activities (UAE-5, 6, 7 and 9), showing clearly that hexane is not a suitable solvent for obtaining extracts with high antiaging potential. One cluster (C3) comprises the extracts with better antioxidant activities and higher TPC (UAE-2, 10 and 12), but low inhibition of elastase and tyrosinase. The extracts included in this cluster have longer extraction times (45 min) and more polar solvents (ethyl acetate and ethanol). Cluster C2 (Figure 1A) is more mixed in terms of solvent, and the main grouping factor is the yield. The extracts included in this cluster were all obtained with a 1:10 solid/liquid ratio, and UAE-8 and 11 presented good tyrosinase inhibition. Cluster C1 (Figure 1A) includes two ethyl acetate extracts obtained after 15 min of extraction (UAE-1 and 3). UAE-1 is the extract with a better yield and better ability to inhibit elastase and tyrosinase. This indicates that ethyl acetate, and 15 min of extraction time are the best conditions to obtain a UAE extract with good antiaging potential.

In the heatmap generated with the MAE data (Figure 1B), the hierarchical clustering led to the formation of five distinct groups. The first cluster (C1) includes extracts with lower yields and poor antiaging activities; namely, all the hexane extracts and the ethanol extract obtained with a shorter time and higher proportion of solvent. The second cluster (C2) includes only MAE-12, which is the extract with a higher yield. However, its antiaging activities are weak, which indicates that inactive compounds were extracted under these conditions. The third cluster (C3), groups the ethyl acetate extracts with a higher antioxidant activities and yield than C1 (Figure 1B), but poor inhibition of elastase and tyrosinase. The fourth cluster (C4) includes only MAE-2, also an ethyl acetate extract, obtained after 20 min and with a ratio of 1:4, which can be differentiated from the other clusters by its higher elastase inhibition, although it has a lower yield. The fifth cluster (C5) aggregates two ethanol extracts (MAE-9 and 10), both obtained with a solid/liquid ratio of 1:4. This cluster suggests that, with MAE, these are the best conditions to extract compounds with high antityrosinase activity, with it being clear that 10 min is enough to extract a high content of polyphenolic compounds with antioxidant activity. Accordingly, ethanol, and a solid–liquid ratio of 1:4 and 10 min are determined as the optimal conditions to prepare an extract with high antiaging potential using MAE as advanced extraction technology.

### 2.3. Factor Analysis of Mixed Data (FAMD)

Although hierarchical clustering has allowed for some conclusions to be reached, only the continuous variables were considered. A further analysis was performed through FAMD to account for the influence of both continuous and categorical variables. The dimensionality reduction achieved through this principal component method is a convenient tool for identifying the relationships amongst variables and hidden patterns in the complex dataset that was obtained. This method was proven to be effective in ecology studies to evaluate compliance with the regulatory framework [40] and develop bioactivity prediction models for fucoidan extracts [41], so it will also be useful to determine the experimental parameters that lead to extracts with higher cosmeceutical potential from UAE and MAE.

#### 2.3.1. Ultrasound-Assisted Extraction 

All the data for the UAE of *Cystoseira abies-marina* were analyzed by FAMD, leading to the generation of dimensions. The associated eigenvalues, variance explained by each dimension and cumulative variance are presented in Table 5.

**Figure 2:**
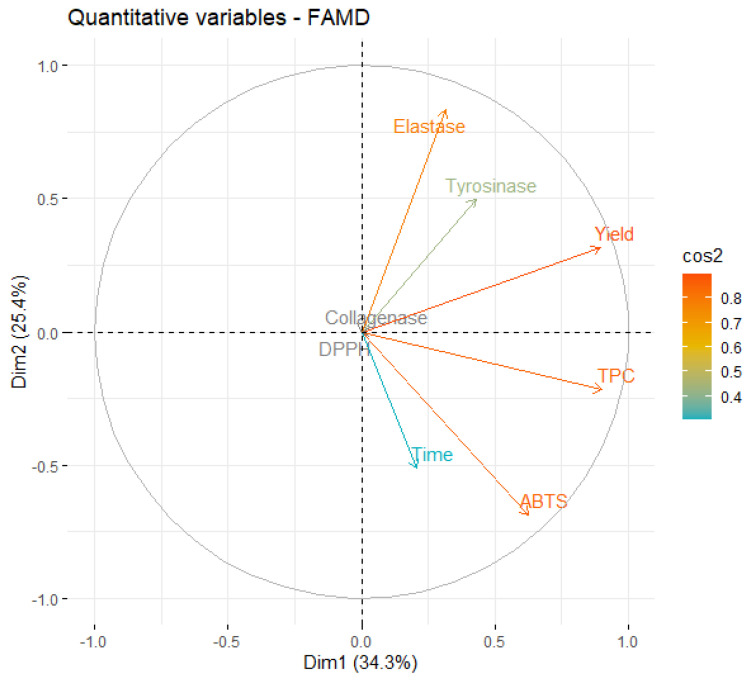
Correlation circle of variables on the first two dimensions of UAE.

An eigenvalue > 1 means that a dimension explains more variance than each of the original variables of the dataset. Specifically, in the case of UAE, the first four dimensions account for more than 86% of the dataset’s variability, showing that this is a fitting model. The first two dimensions explain almost 60% of the variance, so the major focus will be on them. First, a correlation circle was plot (Figure 2), to examine the relationships between the quantitative variables since these correspond to most of the studied variables.

A close analysis of Figure 2 shows that the first two dimensions capture the information contained in most of the quantitative variables, since the individual projection of each variable is very close to the circle. It is also possible to observe that TPC is positively correlated with ABTS (the corresponding vectors point in the same direction of the dimensional space), as described in Section 2.2 and observed in the literature [42,43].

It is also possible to see that an increase in extraction time leads to a higher ABTS activity and TPC, but is negatively correlated with the variables elastase and tyrosinase, meaning that extracts prepared with shorter times are more active against those two enzymes. In practical terms, this may occur because longer extractions lead to an increase in the concentration of other extract components that have higher antioxidant activity but are weaker enzymatic inhibitors. The longer exposure of the extract to ultrasound energy may lead to the degradation of the interest compounds, as described by Babotă et al. [44].

Interestingly, yield is also inversely correlated with time, meaning that better yields are favored by shorter extraction times. This can be considered unexpected, but it has been also reported by other works involving UAE [45]. The positive association of yield with elastase and tyrosinase also indicates that the most active extracts against those enzymes were the ones obtained with higher yields. 

**Figure 3:**
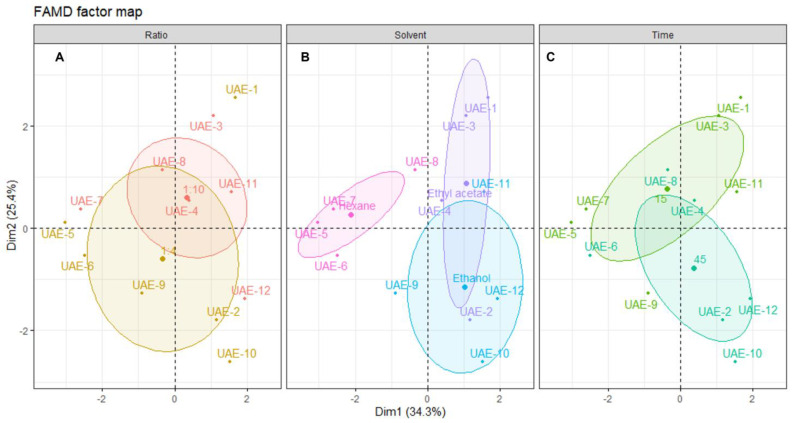
Plot of UAE individuals showing the distribution of samples across the dimensional space. Each panel shows the distribution centered in: (**A**) solid/liquid ratio, (**B**) solvent and (**C**) time of extraction. The shaded ellipsoids depict the 95% confidence ellipsis for outcomes as a function of dimensions 1 and 2.

To better understand the influence of each of the extraction parameters (solvent, s/l ratio and time), the individual extracts were plotted on the dimensional space and grouped according to those parameters (Figure 3).

Analyzing the distribution of each individual sample across the dimensional space and relating this distribution to the correlation circle for quantitative variables shown in Figure 3, it is possible to infer that there is no clear association between the solid/liquid ratio and a particular outcome. As seen in Figure 3A, the confidence ellipsoids for both factors of this variable are highly overlapped, and there are various outliers. The center of the 1:4 ellipsoid is located lower on the dimensional space than the center of the 1:10, which means that higher yields and better activities should be obtained with a ratio of 1:10. However, the observations show that the extract with better yield and inhibitory activities was UAE-1, prepared with a ratio of 1:4, which contributes to the conclusion that this variable is not the most important. From a sustainability perspective, a lower ratio must be chosen to ensure the use of less solvent [46].

Looking at Figure 3B, it is clear that the solvent used in the extraction greatly influences the individual’s position in the dimensional space. Hexane negatively influences the outcome of all other variables, since all individuals are positioned in the inverse direction to the projection of the quantitative variables in Figure 2. This confirms that non-polar solvents are not suitable for obtaining extracts with high antiaging potential from *Cystoseira abies-marina*. The same is not observable on another brown algae species, since the hexane extract of *Sargassum polycystum* was found to be a potent skin-whitening agent, without toxicity for in vitro and in vivo models [47]. Ethanol favors antioxidant activity, especially with a higher extraction time, although there is a lower yield. In turn, ethyl acetate extracts are positioned in the dimensional space towards the projections of yield, tyrosinase and elastase, posing as the most promising solvent for extracts with high antiaging activity. Brown macroalgae extracts prepared with ethyl acetate were reported to present antiaging properties, such as anti-wrinkle properties, found on *Sargassum muticum* [48] or tyrosinase inhibition from *Ecklonia stolonifera* [38]. Note that the extracts of those works were obtained by conventional methods, since there are no records of the use of ethyl acetate on the UAE of macroalgae.

Figure 3C confirms this, showing that higher times are correlated with better ABTS and TPC, and shorter times are more correlated with better yield and enzymatic inhibitory activity. A recent work [49] with the brown macroalgae *Padina pavonica* found no significant difference in the TPC by increasing the extraction time; however, the time was much longer than in the present work. It is very interesting, in terms of sustainability, that shorter extraction times are more suitable for obtaining an extract with higher antiaging activity, since less energy is expended on the process [46]

The FAMD analysis of the UAE data allowed for us to determine that the optimal conditions to obtain an extract with high cosmeceutical potential using this ultrasound energy are ethyl acetate, a solid–liquid ratio of 1:4, and 15 min of extraction. It also showed that it is possible to modulate the extract’s bioactivity according to the intended target by changing the extraction conditions, mostly solvent type and extraction time.

#### 2.3.2. Microwave-Assisted Extraction

The FAMD of the data obtained from the MAE reduced the data to five dimensions, whose respective eigenvalues, % of variance explained by each dimension and cumulative% of variance are presented in Table 6

**Figure 4:**
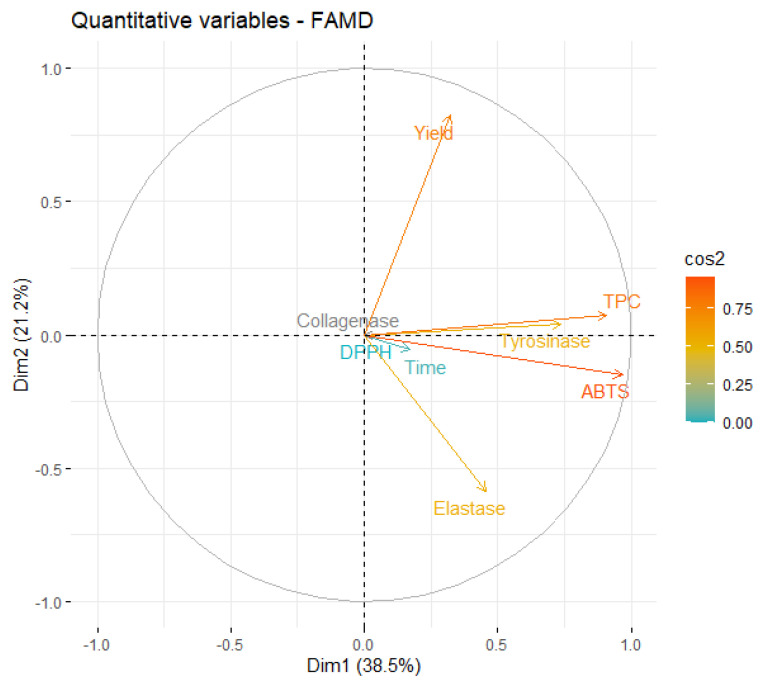
Correlation circle of variables on the first two dimensions of MAE.

Since the first four dimensions have an eigenvalue > 1, they can explain more of the variance than each individual variable of the original dataset. These dimensions account for about 87% of the total variability, which indicates that the FAMD is a fitting model for this analysis. More than half of the variability is explained by the first two dimensions (59.79%), which indicates that the analysis of these dimensions will allow for significant conclusions. A correlation circle is presented in Figure 4 to examine the relationships between the qualitative variables on this dimensional space

From Figure 4, it is possible to conclude that the information contained in most of the quantitative variables is retained in the first two dimensions, since the endpoint of their projection is closer to the circle. The first relationship that must be highlighted is the negative correlation between the yield and inhibition of elastase. However, this correlation is magnified by the fact that, in the original data, the only extract that presented some level of elastase inhibition was obtained in a very low yield.

As expected, ABTS and TPC are highly correlated and, more interestingly, stronger tyrosinase inhibition is also highly correlated with higher phenolic content. In fact, several works found algae polyphenols with anti-tyrosinase activity [39,50], which reinforces our conclusion. 

**Figure 5:**
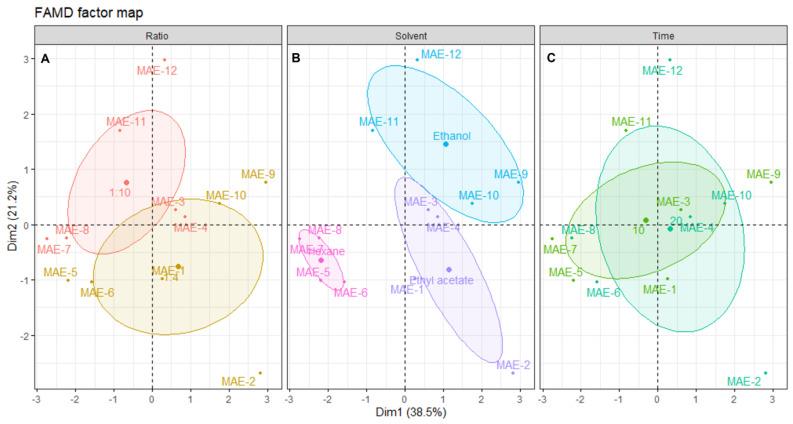
Plot of MAE individuals showing the distribution of samples across the dimensional space. Each panel shows the distribution centered in: (**A**) solid/liquid ratio, (**B**) solvent and (**C**) time of extraction. The shaded ellipsoids depict the 95% confidence ellipsis for outcomes as a function of dimensions 1 and 2.

Further correlations, along with their qualitative parameters, are shown in the plot of individual extracts, along with their distribution across the dimensional space (Figure 5).

From the distribution of each individual extract on the dimensional space and relating this positioning with the correlation circle from Figure 4, it is possible to find a correlation of a solid/liquid ratio with better inhibition of elastase and tyrosinase, and higher antioxidant activity, although better yields are obtained with the 1:10 ratio (Figure 5A). Since this optimization aims to obtain extracts with better antiaging potential, the ratio of 1:4 must be selected, which is also advantageous for sustainability as there is less solvent waste [46].

Figure 5B shows the negative correlation of hexane with all the other variables, mainly yield, tyrosinase inhibition, ABTS and TPC. The bad results obtained with this solvent are mainly attributed to its low dielectric constant, which leads to lower interactions with microwave radiation, and, thus, lower effects of this enhancement technique on the extraction [25]. Ethyl acetate extracts had low activities, except for MAE-2, which was the only extract presenting elastase inhibition and had good antioxidant activity. However, overall, the yields were moderate and the antiaging activity of the other three extracts was low. Ethanol was clearly the best solvent for MAE, not only in terms of yield, but also for antioxidant activities and tyrosinase inhibition. In fact, other works described ethanol as an efficient solvent for obtaining extracts with high antioxidant activity through MAE, although other brown algae were used, such as *Padina pavonica* [49], or *Sargassum muticum* [51], since this is the first study reporting the MAE of *Cystoseira abies-marina*. The higher efficiency of ethanol on MAE might be linked to its higher dielectric constant, and FAMD analysis unequivocally shows that this is the best solvent to obtain extracts with high cosmeceutical potential using this advanced technique.

The extraction time appears to have a low influence on the extraction. In fact, by analyzing Figure 5C, it is possible to observe that the ellipsoids for both factors of this variable are practically overlapping. A recent study about the MAE of polysaccharides from *Cystoseira barbata* and *Fucus virsoides* also found no statistically significant difference when increasing the extraction time [35]. The best yield, elastase inhibition and antioxidant activity were obtained with 20 min of extraction, although the extract with higher activity in terms of the inhibition of tyrosinase was obtained with 10 min of extraction. It is unclear which time should be chosen; however, since the IC_50_ for tyrosinase inhibition of MAE-9 is much lower than the one obtained for elastase by MAE-2, the shorter time should be selected as the one leading to an extract with higher activity. In this regard, it is shown that, despite FAMD allowing for the determination of the ideal conditions for maximum cosmeceutical potential, this also hints at the possibility of modulating the activity by changing the conditions according to the main objective.

## 3. Materials and Methods

### 3.1. Macroalgae Collection

About 3 kg of fresh *Cystoseira abies-marina* (S.G.Gmelin) C.Agardh (reclassified in 2020 as *Gongolaria abies-marina* (S.G.Gmelin) Kuntze) was collected on a rockpool located in Mosteiros (37°53′57″ N, 25°49′18″ W), on the western coast of São Miguel Island, Azores, in July 2020.

An intact specimen was delivered to the Department of Biology of the University of Azores for the specialized identification and creation of vouchers deposited at the Ruy Telles Palhinha Herbarium (SMG-20-23)

After collection, the fresh mass of macroalgae was cleaned to eliminate sand, small rocks, and epiphytes, and was then thoroughly washed with deionized water to remove the excess salts. Then, the material was dried in the dark at room temperature with the aid of a dehumidifier. When all the material was dehydrated, it was ground until a fine powder was obtained.

### 3.2. Extraction of Cystoseira abies-marina

#### 3.2.1. Ultrasound-Assisted Extraction

The dried biomass of *Cystoseira abies-marina* was submitted to different ultrasound-assisted extraction (UAE) conditions, varying the ultrasonication time (15 and 45 min); the solvent type (ethanol, hexane, and ethyl acetate); the algae/solvent ratio (1:4 and 1:10) [52]. The extraction temperature was always maintained below 35 °C (using an ice-bath and a temperature controller). The extraction was performed with a VCX500 ultrasonic processor (VibraCell, Newton, MA, USA, 500 W and 20 kHz) connected to a probe tip NO. 630-0220 with 13 mm diameter. The extraction yield was obtained as follows:

Y_ext_ = (m_ext_/m_alg_)

where m_ext_ is the mass (mg) of dried extract and m_alg_ corresponds to the mass (g) of dried algae used to obtain the extract. Results were expressed in mg extract/ g d.w.

#### 3.2.2. Microwave-Assisted Extraction

Several *Cystoseira abies-marina* extracts were prepared on an Ethos SYNTH microwave apparatus (Milestone Inc., Sorisole (BG), Italy), varying the irradiation time (10 and 20 min), the solvent (ethanol, hexane, and ethyl acetate), and algae/solvent ratio (1:4 and 1:10). The potency defined was 40 W, and extraction time was adjusted to avoid the temperature rising above 35 °C. The extraction yield was obtained as described above.

### 3.3. Biological Activities

#### 3.3.1. DPPH Radical Scavenging Activity

Antioxidant activity was assayed by the 1,1-diphenyl-2-picryl-hydrazyl (DPPH) radical scavenging assay [53]. Serial dilutions of studied extracts, or reference compound (Trolox) were carried out in 96-well microplates, with concentrations ranging between 0.244 μg/mL and 250 μg/mL in methanol. DPPH dissolved in methanol was added to the microwells, yielding a final concentration of 45 µg/mL, and the absorbance at 515 nm was measured with a BioRad Microplate Reader Model 680 (Bio-Rad Laboratories, Inc., Hercules, CA, USA), after 30 min in the dark. In each assay, a control was prepared, in which the same amount of solvent substituted the sample or standard. Percentage of antioxidant activity (% AA) was calculated as:

%AA = [(Abs_control_ − Abs_sample_)]/(Abs_control_)] × 100

where Abs_control_ is the absorbance of the control and Abs_sample_ is the absorbance of the alga extract or standard. All assays were carried out in triplicate and the results expressed as IC_50_, i.e., as the concentration yielding 50% scavenging of DPPH, calculated by interpolation from the% AA vs. concentration curve.

#### 3.3.2. ABTS Radical Scavenging Assay

The method of Re et al. [54] was adopted to perform ABTS radical scavenging assay. The stock solutions included a 7 mM ABTS solution (2,2′-azinobis-(3 ethylbenzothiazoline 6 sulfonic acid)) and a 2.4 mM potassium persulfate solution. The working solution was prepared by mixing the two stock solutions in equal quantities and allowing them to react for 12–16 h at room temperature in the dark. The solution was then diluted by mixing 1 mL ABTS solution with the amount of methanol necessary to obtain an absorbance of 0.7 at 734 nm. Serial dilutions of studied extracts or reference compound (Trolox) were carried out in 96-well microplates, with concentrations ranging between 0.244 μg/mL and 250 μg/mL in methanol. ABTS solution was then added to the microwells, and after 8 min of incubation the absorbance was recorded at 750 nm with a BioRad Microplate Reader Model 680 (Bio-Rad Laboratories, Inc., Hercules, CA, USA). In each assay, a negative control was prepared, in which the same amount of solvent substituted the sample. The percentage of antioxidant activity (% AA) was calculated as:

%AA = [(Abs_control_ − Abs_sample_)]/(Abs_control_)] × 100

where Abs_control_ is the absorbance of ABTS radical + methanol; Abs_sample_ is the absorbance of ABTS radical + sample/standard. 

All assays were carried out in triplicate and results expressed as IC_50_, i.e., as the concentration yielding 50% scavenging of ABTS, calculated by interpolation from the% AA vs. concentration curve.

#### 3.3.3. Total Phenolic Content (TPC) Determination

Total phenolic content of the extracts was determined by the Folin-Ciocalteau method described by [55], adapted to microscale. In brief, 5 μL of each extract at a concentration of 50 mg/mL was mixed with 50 μL of distilled water and 100 μL of 10% Folin–Ciocalteu reagent. The solution was stirred and after 3 min, 200 μL 10% sodium carbonate solution was added. Samples were left in the dark at room temperature for 1 h. The absorbance was read at 750 nm with a BioRad Microplate Reader Model 680 (Bio-Rad Laboratories, Inc., Hercules, CA, USA). The same process was repeated for gallic acid at final concentrations ranging from 0 to 500 μg/mL, and the obtained values were used to plot a calibration curve. TPC of the extracts was obtained by interpolation from the gallic acid calibration curve, and results were expressed as gallic acid equivalents in μg/mg of dried extract (μg GAE/mg).

#### 3.3.4. Hyaluronidase Inhibition Assay

The methods described by Ndlovu et al. [56] and Zhou et al. [57] were applied for the hyaluronidase inhibition assay. The following was added into 2 mL test tubes: 50 μL of calcium chloride (12.5 mM), 50 μL of test samples or sodium aurothiomalate diluted in 100 mM acetate buffer, pH 3.5 (with concentrations ranging between 15.6–250 μg/mL), and 25 μL hyaluronidase (0.5 mg/mL). The tubes were incubated in a water bath (37 °C; 20 min), after which 50 μL of the substrate hyaluronic acid (0.25 mg/mL in 100 mM acetate buffer, pH 3.5) was added and the tubes incubated for further 40 min. A volume of 25 µL of KBO_2_ 4H_2_O (800 mM) was added to all tubes, which were placed in a water bath (100 °C) for 3 min and left to cool to room temperature; then, and 800 μL of DMAB (4-dimethylaminobenzaldehyde) (4 g DMAB in 40 mL acetic acid and 5 mL 10 N HCl) was added. The tubes were then incubated for 20 min and the contents transferred to respective wells in a 96-well plate. Absorbance was detected at 585 nm Bio Rad Model 680 Microplate Reader (Bio-Rad Laboratories, Inc., Hercules, CA, USA). Percentage of hyaluronidase inhibition was calculated as:

% Hyaluronidase inhibition = [(Abs_control_ − Abs_sample_)]/(Abs_control_)] × 100

where Abs_control_ is the absorbance of buffer + hyaluronidase; Abs_sample_ is the absorbance of buffer + hyaluronidase + sample/standard. 

All assays were carried out in triplicate and results expressed as IC_50_, i.e., as the concentration yielding 50% of hyaluronidase inhibition, calculated by interpolation from the% hyaluronidase inhibition vs. concentration curve.

#### 3.3.5. Tyrosinase Inhibition Assay

The extracts were assayed by adapting the tyrosinase inhibition method described by Shimizu et al. [58] and modified by Manosroi et al. [59]. In brief, 25 μL of tyrosinase enzyme solution (135 U/mL), 25 μL of ten serial concentrations of the extracts (0.488 μg/mL to 250 μg/mL dissolved in 100 mM phosphate buffer, pH 6.8 containing no more than 2.5% DMSO), and 100 μL phosphate buffer were mixed in a 96-well plate, and incubated at 37 °C for 20 min. Then, 50 μL of 1.66 mM of tyrosine solution in 100 mM phosphate buffer, pH 6.8, was added. The enzyme activity was measured at 490 nm every 10 min for 30 min in a Bio Rad Model 680 Microplate Reader (Bio-Rad Laboratories, Inc., Hercules, CA, USA). Kojic acid at 0.293–150 μg/mL was used as a positive control. The experiments were carried out in triplicate. For each concentration, enzyme activity was calculated as a percentage of the velocities compared to that of the assay using buffer without any inhibitor. The IC_50_ value, which was the sample concentration that inhibited 50% of the enzyme activity, was determined by interpolation from the% tyrosinase inhibition vs. concentration curve.

#### 3.3.6. Elastase Inhibition Assay

The extracts were assayed by the method described by Ndlovu et al. [56] with some modifications. In brief, 25 μL of elastase enzyme solution (0.3 U/mL), 50 μL of ten serial concentrations of the extracts or fractions (0.488 μg/mL to 250 μg/mL dissolved in 100 mM HEPES buffer, pH 7.5 containing no more than 2.5% DMSO) and 125 μL HEPES buffer were mixed in a 96-well plate and incubated at room temperature for 20 min. Then, 50 μL of *N*-methoxysuccinyl-Ala-Ala-Pro-Val-p-nitroanilide (1 mM) was added. The enzyme activity was measured at 405 nm in the moment of substrate addition and after 40 min of incubation at 25 °C in a Bio Rad Model 680 Microplate Reader (Bio-Rad Laboratories, Inc., Hercules, CA, USA). *N*-Methoxysuccinyl-Ala-Ala-Pro-chloromethyl ketone at 0.019–20 μg/mL was used as a positive control. The experiments were carried out in triplicate. For each concentration, enzyme activity was calculated as a percentage of the velocities compared to that of the assay, using buffer without any inhibitor. The IC_50_ value, which was the sample concentration that inhibited 50% of the enzyme activity, was determined interpolation from the% elastase inhibition vs. concentration curve.

#### 3.3.7. Collagenase Inhibition Assay

An adaptation of the method of Mandl et al. [60] was used to determine anti-collagenase activity. The following was added to 2 mL test tubes: 25 μL of collagenase solution (0.8 U/mL), 25 μL TES buffer (50 mM) with 0.36 mM calcium chloride, pH 7.4 and 50 μL of test sample or the reference compound EDTA (with concentrations ranging between 15.6 and 250 μg/mL). The tubes were incubated in a water bath at 37 °C for 20 min. Thereafter, 50 μL FALGPA (1 mM) solution was added to the tubes and further incubated for 60 min at 37 °C. To all tubes, 200 μL of a solution containing equal volumes of a 1.6 mg/mL Tin chloride (II) solution in 200 mM citrate buffer, pH 5, and 50 mg/mL ninhydrin solution in DMSO was added. All tubes were placed in a water bath (100 °C) for 5 min and left to cool to room temperature before adding 200 μL of 50% isopropanol to each tube. Contents in the tubes were then transferred to respective wells in 96-well plates. Absorbance was detected at 550 nm Bio Rad Model 680 Microplate Reader (Bio-Rad Laboratories, Inc., Hercules, CA, USA). Percentage of collagenase inhibition was calculated as:

% Collagenase inhibition = [(Abs_control_ − Abs_sample_)]/(Abs_control_)] × 100

where Abs_control_ is the absorbance of buffer+collagenase; Abs_sample_ is the absorbance of buffer + collagenase + sample/standard. 

All assays were carried out in triplicate and results expressed as IC_50_, i.e., as the concentration yielding 50% of collagenase inhibition, calculated by interpolation from the% collagenase inhibition vs. concentration curve.

### 3.4. Statistical Analysis

A two-way ANOVA, followed by post-hoc HSD Tukey’s test, were used to assess the significant differences between samples in each assayed biological test, using the open-source software R [61] (4.2.1 for Windows) and RStudio.

Each variable was autoscaled (*z*-score calculation) and the data was visualized through a heatmap combined with hierarchical clustering, through the functions provided by the R package “pheatmap” [62]. The data were further analyzed by factor analysis on mixed data (FAMD), using the packages “FactoMineR” [63] and “factoextra” [64].

## 4. Conclusions

The present work described a new approach to optimizing the extraction parameters of both ultrasound- and microwave-assisted extraction, which consisted of finding the parameters which increase the antiaging activities of *Cystoseira abies-marina* extracts (bio-guided optimization).

A thorough statistical analysis of the data obtained, enabled the determination of the ideal conditions for enhanced cosmeceutical potential. Regarding UAE, those conditions were ethyl acetate, solid/liquid ratio of 1:4 and 15 min of extraction, which led to extracts with high yield and good tyrosinase and elastase inhibition. For MAE, the best conditions were ethanol, solid/liquid ratio of 1:4 and 10 min of extraction, producing extracts with better antioxidant activity, moderate yield, and tyrosinase inhibition. The differences between the results obtained for each extraction method demonstrate that, depending on the application of the extract, its activity might be modulated by changing the extraction method and the parameters, which is an advantage, since the same algal material can originate products with distinct cosmeceutical application.

The extraction parameters determined as the best for each technique also ensure the method’s sustainability due to the lower solvent and energy consumption, demonstrating the potential for industrial applications. 

To the best of our knowledge, this is the first report about the antiaging activities of *Cystoseira abies-marina* and the extraction of this biological matrix using UAE and MAE. A scale-up production and full extract characterization is ongoing, as a proof-of-concept to further explore the potential of these extracts for cosmeceutical applications.

## Figures and Tables

**Table 1:** Extraction yields of *Cystoseira abies-marina* UAE obtained with different experimental parameters.

Sample	Solvent	s/l Ratio *	Time (min)	Extract Yield (mg Extract/g d.w) **
UAE-1	Ethyl Acetate	1:4	15	10.0 ^a^
UAE-2	Ethyl Acetate	1:4	45	6.5^b^
UAE-3	Ethyl Acetate	1:10	15	6.6 ^b^
UAE-4	Ethyl Acetate	1:10	45	8.3 ^c^
UAE-5	Hexane	1:4	15	0.9 ^d^
UAE-6	Hexane	1:4	45	0.7 ^d^
UAE-7	Hexane	1:10	15	1.4 ^e^
UAE-8	Hexane	1:10	45	6.9 ^f^
UAE-9	Ethanol	1:4	15	3.0 ^g^
UAE-10	Ethanol	1:4	45	5.0 ^h^
UAE-11	Ethanol	1:10	15	8.2 ^i^
UAE-12	Ethanol	1:10	45	8.6 ^j^

Temperature was maintained below 35 °C; * solid/liquid ratio; ** d.w—dry weight; different letters indicate significant differences (*p* < 0.05).

**Table 2:** Extraction yields of *Cystoseira abies-marina* MAE obtained with different experimental parameters.

Sample	Solvent	s/l Ratio *	Time (min)	Extract Yield (mg Extract/g d.w) **
MAE-1	Ethyl Acetate	1:4	10	0.81 ^a^
MAE-2	Ethyl Acetate	1:4	20	0.78 ^b^
MAE-3	Ethyl Acetate	1:10	10	2.2 ^c^
MAE-4	Ethyl Acetate	1:10	20	2.1 ^d^
MAE-5	Hexane	1:4	10	0.14 ^e^
MAE-6	Hexane	1:4	20	0.19 ^f^
MAE-7	Hexane	1:10	10	0.06 ^g^
MAE-8	Hexane	1:10	20	0.26 ^h^
MAE-9	Ethanol	1:4	10	2.6 ^i^
MAE-10	Ethanol	1:4	20	1.6 ^j^
MAE-11	Ethanol	1:10	10	2.8 ^k^
MAE-12	Ethanol	1:10	20	7.8 ^l^

Temperature was maintained below 35 °C; * solid/liquid ratio; ** d.w—dry weight; different letters indicate significant differences (*p* < 0.05).

**Table 3:** Antioxidant activities of the extracts of *Cystoseira abies-marina*.

Sample	DPPH		ABTS		TPC
	% AA *	EC_50_ µg/mL	% AA *	EC_50_ µg/mL	μg GAE/mg **
UAE-1	33.5 ± 0.41	>250 ^a^	58.6 ± 0.39	236.1 ± 0.12 ^a^	13.24 ± 0.05 ^a^
UAE-2	40.2 ± 0.55	>250 ^a^	68.2 ± 0.37	191.1 ± 0.88 ^b^	17.31 ± 0.31 ^b^
UAE-3	32.6 ± 0.74	>250 ^a^	56.8 ± 0.89	244.1 ± 0.61 ^c^	15.89 ± 0.15 ^c^
UAE-4	11.9 ± 0.99	>250 ^a^	37.9 ± 0.12	>250 ^d^	9.87 ± 0.02 ^d^
UAE-5	0	>250 ^a^	0	>250 ^d^	4.65 ± 0.03 ^e^
UAE-6	0	>250 ^a^	0	>250 ^d^	5.25 ± 0.01 ^f^
UAE-7	0	>250 ^a^	10.1 ± 0.50	>250 ^d^	5.55 ± 0.02 ^g^
UAE-8	36.8 ± 1.01	>250 ^a^	46.1 ± 0.98	>250 ^d^	9.90 ± 0.07 ^d^
UAE-9	40.7 ± 0.15	>250 ^a^	56.9 ± 0.63	240.5 ± 0.39 ^e^	12.13 ± 0.01 ^h^
UAE-10	33.8 ± 0.69	>250 ^a^	61.2 ± 0.87	180.2 ± 0.85 ^f^	18.96 ± 0.09 ^i^
UAE-11	34.5 ± 0.21	>250 ^a^	58.5 ± 0.84	233.3 ± 0.55 ^g^	16.71 ± 0.15 ^j^
UAE-12	29.2 ± 0.86	>250 ^a^	61.0 ± 0.96	182.1 ± 0.99 ^h^	14.02 ± 0.02 ^k^
MAE-1	16.44 ± 0.46	>250 ^a^	63.04 ± 1.0	168.74 ± 1.4 ^i^	6.82 ± 0.06 ^l^
MAE-2	26.00 ± 0.80	>250 ^a^	77.45 ± 0.94	89.40 ± 0.62 ^j^	9.89 ± 0.20 ^d^
MAE-3	15.73 ± 0.18	>250 ^a^	63.73 ± 1.6	160.69 ± 0.65 ^k^	11.05 ± 0.05 ^m^
MAE-4	15.18 ± 0.76	>250 ^a^	61.10 ± 0.64	164.83 ± 0.66 ^l^	8.68 ± 0.11 ^n^
MAE-5	0	>250 ^a^	14.37 ± 0.56	>250 ^d^	1.22 ± 0.05 ^o^
MAE-6	0	>250 ^a^	14.33 ± 0.90	>250 ^d^	4.51 ± 0.01 ^e^
MAE-7	0	>250 ^a^	10.67 ± 0.30	>250 ^d^	0 ^g^
MAE-8	0	>250 ^a^	13.15 ± 0.45	>250 ^d^	2.37 ± 0.01 ^q^
MAE-9	22.18 ± 0.53	>250 ^a^	75.72 ± 0.63	96.77 ± 0.44 ^m^	13.03 ± 0.01 ^h^
MAE-10	14.65 ± 0.28	>250 ^a^	60.65 ± 1.7	143.07 ± 0.79 ^n^	7.05 ± 0.01 ^l^
MAE-11	6.95 ± 0.18	>250 ^a^	33.93 ± 0.97	>250 ^d^	5.81 ± 0.02 ^gr^
MAE-12	11.74 ± 0.23	>250 ^a^	52.63 ± 1.5	191.86 ± 1.1 ^b^	5.99 ± 0.03 ^r^
Trolox	89.7 ± 0.50	7.3 ± 0.09 ^b^	87.1 ± 0.95	2.68 ± 0.08 ^o^	-

* % Antioxidant activity at 250 µg/mL; ** μg Gallic acid equivalents (GAE)/mg of dried extract; In each column, different letters indicate significant differences (*p* < 0.05).

**Table 4:** Antiaging activities of the *Cystoseira abies-marina* extracts.

Sample	Elastase		Tyrosinase		Collagenase
	% Inhibition *	IC_50_ µg/mL	% Inhibition *	IC_50_ µg/mL	% Inhibition *
UAE-1	92.1 ± 0.02	45.4 ± 0.14 ^a^	90.1 ± 0.11	7.5 ± 0.43 ^a^	49.8 ± 0.12
UAE-2	23.2 ± 0.19	>250 ^b^	52.5 ± 0.58	241.6 ± 0.14 ^b^	0
UAE-3	92.1 ± 0.95	65.1 ± 0.41 ^c^	68.0 ± 0.82	143.5 ± 0.91 ^c^	0
UAE-4	56.9 ± 0.37	231.6 ± 0.12 ^d^	65.6 ± 0.75	156.8 ± 0.48 ^d^	0
UAE-5	62.8 ± 0.71	184.6 ± 0.43 ^e^	0	>250 ^e^	33.3 ± 0.77
UAE-6	0	>250 ^b^	73.1 ± 0.88	99.8 ± 0.65 ^f^	36.7 ± 0.14
UAE-7	0	>250 ^b^	60.0 ± 1.49	185.1 ± 0.69 ^g^	21.7 ± 0.23
UAE-8	68.6 ± 0.28	178.8 ± 0.85 ^f^	85.5 ± 0.62	23.4 ± 0.21 ^h^	12.5 ± 0.66
UAE-9	0	>250 ^b^	0	>250 ^e^	0
UAE-10	0	>250 ^b^	68.2 ± 0.14	140.1 ± 0.11 ^i^	11.9 ± 0.64
UAE-11	60.3 ± 0.52	186.5 ± 0.99 ^g^	80.2 ± 1.6	29.7 ± 0.98 ^j^	0
UAE-12	0	>250 ^b^	73.5 ± 0.36	101.2 ± 0.44 ^k^	16.2 ± 0.91
MAE-1	40.5 ± 0.31	>250 ^b^	47.4 ± 0.23	>250 ^e^	7.9 ± 0.35
MAE-2	56.6 ± 1.23	220.8 ± 0.87 ^h^	65.9 ± 0.73	179.8 ± 0.31 ^l^	22.3 ± 0.69
MAE-3	37.9 ± 0.52	>250 ^b^	49.4 ± 0.57	>250 ^e^	11.2 ± 0.99
MAE-4	45.7 ± 0.68	>250 ^b^	67.8 ± 0.20	175.6 ± 0.47 ^m^	27.3 ± 0.87
MAE-5	39.2 ± 1.99	>250 ^b^	0	>250 ^e^	9.5 ± 0.26
MAE-6	41.8 ± 1.20	>250 ^b^	0	>250 ^e^	26.9 ± 0.37
MAE-7	33.3 ± 0.48	>250 ^b^	0	>250 ^e^	21.8 ± 0.84
MAE-8	28.1 ± 0.56	>250 ^b^	0	>250 ^e^	5.6 ± 0.64
MAE-9	44.7 ± 0.96	>250 ^b^	93.2 ± 0.72	42.6 ± 0.80 ^n^	16.0 ± 0.24
MAE-10	26.8 ± 0.40	>250 ^b^	88.2 ± 0.15	65.4 ± 0.33 ^o^	21.2 ± 0.56
MAE-11	19.7 ± 0.51	>250 ^b^	30.2 ± 0.81	>250 ^e^	13.8 ± 0.56
MAE-12	40.0 ± 0.22	>250 ^b^	44.1 ± 51	>250 ^e^	15.1 ± 0.24
NMAAK **	95.5 ± 3.96	0.13 ± 0.002 ^i^	-	-	-
Kojic Acid	-	-	99.2 ± 0.36	1.82 ± 0.13 ^p^	-
EDTA	-	-	-	-	96.3 ± 1.2

* % inhibitory activity at 250 µg/mL; ** *N*-Methoxysucinil-Ala-Ala-Pro-Val-chloromethylketone; In each column, different letters indicate significant differences (*p* < 0.05).

**Table 5:** Eigenvalue and variability explained by the FAMD model for UAE.

Dimension	Eigenvalue	% Variance	Cumulative% Variance
Dim. 1	3.086	34.29	34.29
Dim. 2	2.289	25.43	59.72
Dim. 3	1.348	14.98	74.70
Dim. 4	1.029	11.43	86.13
Dim. 5	0.732	8.13	94.26

**Table 6:** Eigenvalue and variability explained by the FAMD model for MAE.

Dimension	Eigenvalue	% Variance	Cumulative% Variance
Dim. 1	3.47	38.54	38.54
Dim. 2	1.91	21.24	59.79
Dim. 3	1.35	15.05	74.84
Dim. 4	1.13	12.60	87.44
Dim. 5	0.51	5.71	93.15

## Data Availability

The data presented on this study are available in the article.
